# The regional differences in prevalence, medical expenditures and risk factors for injury in Taiwanese teenagers

**DOI:** 10.1186/1471-2458-6-107

**Published:** 2006-04-26

**Authors:** Huei-Yang Chen, Hsing-Yi Chang, Shu-Fang Shih, Chih-Cheng Hsu, Yu-Hsuan Lin, Yaw-Tang Shih

**Affiliations:** 1 Center for Health Policy Research and Development, National Health Research Institutes, No. 35, Keyan Road, Zhunan Town, Miaoli County 350, R.O.C, Taiwan; 2 Center for Population and Health Survey Research, Bureau of Health Promotion, Department of Health, 5F, No.503, Sec. 2, Liming Road, Xitun District, Taichung City 408, R.O.C, Taiwan

## Abstract

**Background:**

Injury is the leading cause of death in teenagers worldwide. In Taiwan, people in mountainous areas have a 4 to 8 years shorter life span than the general population. Injury among teenagers is likely a major cause. The objective of this study was to investigate the regional differences in the prevalence, the risk factors, and the medical expenditures for injury among Taiwanese teenagers.

**Method:**

An equal probability national sample was used. In addition, representative samples from mountainous areas and offshore islands were used. Only those who aged between 12 and 21 years, and signed the consent form permitting us to link their National Health Insurance (NHI) claim data were included in the analysis. Injury-related visits and expenditures in outpatient services were extracted from the NHI data. Logistic regression was used to examine the factors associated with injury. For those who had injury related outpatient visits, mixed model was used to examine the factors associated with medical expenditures accounting for multiple visits by the same individual.

**Results:**

The prevalence of nonfatal injury was around 30% of teenagers in Taiwan. It was 10% higher in mountainous areas. Factors associated with injury were those who lived in mountainous areas (adjusted odds ratio [OR]: 1.7; 95%; confidence interval [CI]: 1.3–2.3), males (OR: 1.3; 95%; CI: 1.1–1.6), older teens (18–21 years old), and those with risk behavior were positively associated with injury. These factors were also associated with the number of injury-related outpatient visits. However, teenagers in mountainous areas did not spend more on medical care than those who lived in metropolitan Taiwan.

**Conclusion:**

Around 30% of the teenagers were injured in a year, not including the dead. Each of the injured spent at least 851.4NTD (~27USD) for outpatient visits. The scope of the problem was not trivial. Hazardous environments and high-risk behaviors were the universal causes. In remote areas, lack of medical resources was another possibility. Empowering local people to design prevention programs according to their needs is recommended.

## Background

Injuries accounted for 9% of the world's deaths and 12% of the world's burden of disease in 2000 [1]. Although most injuries are preventable, it is expected to rise dramatically by 2020 [2]. However, the probability of injury is unequally distributed among all groups in a society. It can be affected by factors that have been observed in previous studies, such as gender, age, socioeconomic status, and health behaviors [3-5]. Although mortality rate of accidental injury among Taiwanese teenagers showed a slight decrease, fell to 48.2 deaths per 100,000 males in 2002; it was still higher compared to the rest of Asia, with the exception of South Korea. Furthermore, the teenage females' injury mortality rate in Taiwan was the highest in Asia (the same as South Korea), with 19.5 deaths per 100,000 [6].

Central Taiwan is covered by mountains, which account for three-fifths of the whole area. More than 100 mountains are higher than 3000 m (~12,000ft.). The Taiwan government classified 30 townships as mountainous. Approximately 80% of the residents of these counties were Taiwanese aborigines. Wen et al. compared the life expectancies between people living in mountainous areas and those living in the general area of Taiwan between years 1970 and 2000, and found that the life expectancy was 8.5 years shorter for males from 1971 to 1973; from 1998 to 2000 the expectancy was 13.5 years shorter. The life-expectancy gap for females remained relatively stable, about 8 years during study period [7]. Hurng and colleagues conducted a study in aboriginal townships of Taiwan and found that the most common health problems were accidents, hypertensive diseases, tuberculosis, drinking problem and betel nuts chewing [8].

According to World Health Organization (WHO) statistics, injury was the leading cause of death worldwide in those younger than 24 years old [1]. The same finding was reported in Taiwan [6]. Based on the official statistics, the mortality rate of accidental injury in teenagers of mountainous areas was higher than that in other areas of Taiwan and offshore islands, and the rate was increasing with age regardless of area. Specifically, the mortality rate of mountainous teenagers was about three times higher than the overall rate of Taiwan. (Calculated by death registry and population by age and county from Ministry of the Interior, Taiwan, R.O.C.) [9,10]. Therefore, the shorter life span of those in mountainous areas was worthy of in-depth investigation. The injury rate among teenagers was likely a major cause of the difference in life expectancy. It was also worth investigating which risk factors were associated with injury.

Injuries not only cause the most deaths in teenagers, but also require substantial medical resources [11]. For example, the cost of road injuries in some low- and middle-income areas exceeded what they received in economic development assistance [12]. However, few studies have explored the prevalence and cost of teenagers' injury, and even fewer have studied the impact of regional differences. Therefore, this observational study aimed to investigate the prevalence of teenagers' injuries, the expenditures for medical care, and the risk factors associated with various regions of Taiwan. This may provide justification for enforcing injury prevention programs targeting the high-risk groups.

## Methods

### Sample

Study subjects were Taiwan residents aged between 12 and 21 years old, who participated the National Health Interview Survey (NHIS), which was conducted by the Division of Health Policy Research, National Health Research Institutes and the Bureau of Health Promotion of Department of Health (DOH) in 2001 [13]. This survey incorporated a multi-stage stratified systematic sampling scheme. It divided 359 townships or districts of Taiwan into seven strata according to their geographic locations and degrees of urbanization. Townships or districts in each stratum were selected with probability proportional to sizes (PPS). In each selected township or district, lins (the smallest administrative unit) were selected with PPS. Four households were selected randomly from each selected lin. All household members were interviewed. Altogether, 6,592 households (26,658 persons) were sampled to represent the general area of Taiwan. Additional samples were independently selected from mountainous areas and offshore islands because of their small populations. In mountainous and offshore areas, 608 households (2,797 persons) and 432 households (1,954 persons) were sampled, respectively. This resulted in equal probability samples [15].

The final version of the questionnaire comprised five parts: questions for (1) households, (2) individuals older than 12 years, (3) individuals younger than 12 years, (4) a self-administered questionnaire for teenagers aged between 12 and 19 years old, and (5) WHO quality of life (BRIEF) for adults aged between 20 and 65 years old. The response rate was 91.1% for households and 94.2% for individuals. About 86% of the respondents signed a consent form to permit us access to their medical claim data from the Bureau of National Health Insurance. A detail design of the survey has been reported elsewhere [13,14]. Three independent samples for general area of Taiwan, mountainous areas, and offshore islands were used in this study.

After accessing the respondents' NHI claim data, we identified the injury-related outpatient visits by ICD-9-CM. The ICD-9-CM was based on the "ICD_9_CM Millennium Edition International Classification of Diseases 9th Revision Clinical Modification". There were some A-codes in the NHI data that we counted according to the standard published by the Taiwan Hospital Association. The injuries definition followed the classification in other studies like Mo in Canada [15]. The five categories were: (1) Open Wounds (8700–8842 and 8900–8942); (2) Fractures (8000–8291); (3) Contusions (9200–9249); (4) Strains/Sprains (8400–8489); and (5) Other Injuries (8300–8399, 8500–8541, 8600–8691, 9000–9199, and 9300–9999).

The injury prevalence and related expenditures for outpatient services were investigated. There were 61 boys and 79 girls hospitalized. Only 7 and 16 people were hospitalized in offshore and mountainous areas respectively. We did not consider hospitalization in the study, due to the small sizes in some cells. Demographic characteristics, which included gender, age groups and resident area, were considered as predisposing factors that associated with injury. Health behaviors were considered as enabling factors in the model.

Teenagers were categorized into three age groups (12–14, 15–17, and 18–21 years old). An indicator was used to represent the location of the teenagers. Household income was used to represent socioeconomic status. In Taiwan, it is the obligation and right for everyone to get education up to junior high school. This survey reported over 95% of teenagers attended school for at least 9 years. According to the 12–19 years old self-administrated questionnaire, some of teens in mountainous areas went to work or did part-time job after 15 years old. However, the year of education was strongly associated with teens' age (χ^2^<0.0001). Therefore, education levels were excluded from the analysis to avoid co-linearity with age. Health behaviors such as smoking, drinking, chewing betel nut, and using protective devices while driving or riding, were used in the model. The responses of substance use were divided into "yes" or "no," since as teenagers they were starting to develop habits. Due to high correlation in the use of substances, we combined smoking, drinking, and chewing betel nuts into one variable, which contained three categories: no risky behavior, one risky behavior, and two or more risky behaviors. The types of injury were included in the analysis of injury-related cost adjusting the difference of cost. The commonest injury, Sprains/Strains, which was stable in three areas, was used as reference.

### Statistical analysis

We used SAS software version 8 to conduct all statistical analyses [16]. We used the log-linear model to compare the characteristics of nonfatal injuries. Then, the concept of two part model was applied. In the first part, logistic regression was used to investigate the chance of injury given the possible risk factors. In the second part, we examined the factors associated with number of outpatient visits or the cost for the injured. We categorized injury-related visits into three levels: zero, once, and twice or more in one year. We used the proportional odds model to examine the factors for injury-related visits. The proportional odds model has been used whenever the response variable has more than two levels and ordinal.

The model may be represented by a series of logistic regressions for dependent binary variables, with common regression parameters reflecting the proportional odds assumption. We used the mixed model to analyze logarithmic cost in the injured group, accounting for multiple types of injuries of the same person. Injury-related visits for the same type of injury in outpatient services were summed for each individual. Since the injury-related cost was skewed, logarithm transformation was used to make the dependent variable normally distributed.

## Results

A total of 3,152 teenagers were interviewed and signed the NHIS consent form. The sample size from the overall area of Taiwan was 2,755; from mountainous regions, 220; and from offshore areas 177 persons. We presented the injury prevalence by regions and the other predisposing variables in Table 1.

The results showed that teenagers lived in mountainous areas had the highest injury prevalence (40.9%; adjusted residual: 3.4). The prevalence of nonfatal injuries increased with age (12–14 years old: 22.6%; 15–17 years old: 27.9%; 18–21 years old: 35.6%). It was higher in young men than in young women (Males: 34.7%; Females: 26.7%). Teens in senior high school had the highest injury prevalence (35.1%; adjusted residual: 4.7). We found no difference of injury by household income levels. Teens with one or more risk behaviors had significantly higher prevalence of injury than those reported no risk behavior, (one: 42.9%; two or more: 44.8%). Finally, those who used protective devices had higher injury prevalence than their counterparts.

**Figure 1:**
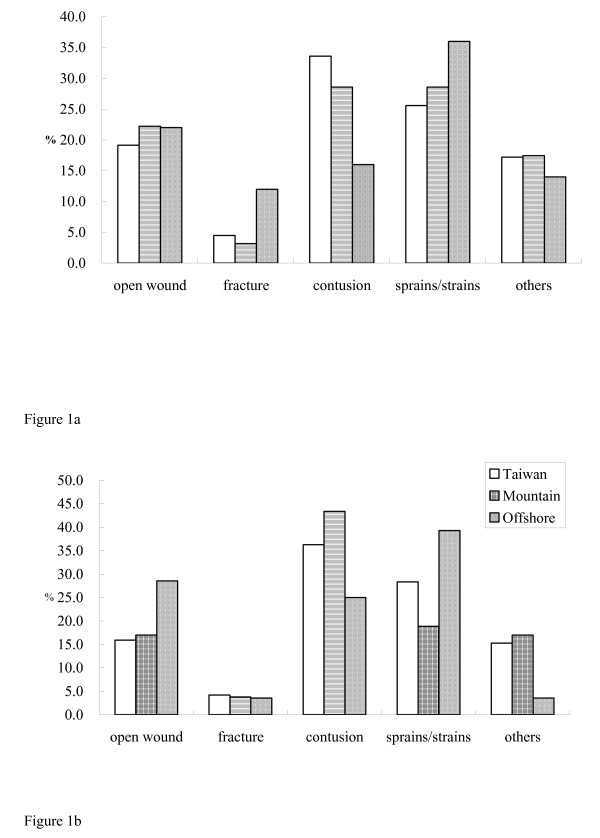
Figure 1a: Distribution of injury types in various regions for Taiwanese males. Figure 1b: Distribution of injury types in various regions for Taiwanese females

Figure 1 shows the distribution of injury types. In Taiwan, the most common injury was contusions, followed by sprains/strains, and open wounds regardless of gender (Fig 1). Males in remote areas had a slightly higher proportion of open wounds and sprains/strains than the males of Taiwan. In females, the proportions of open wounds and sprains/strains of those in offshore areas were higher than those in other areas, and the proportion of contusions was the highest for those in mountainous areas.

The results of logistic regression showed that people in mountainous areas had 1.7 (95% confidence interval [CI]: 1.3–2.3) times higher chance of injury than their counterparts in Taiwan (Table 2). Males were more likely to be injured than females (odds ratio [OR]: 1.3; 95% CI: 1.1–1.6). The younger age groups had a lower probability of being injured than the oldest group. (OR: 0.6; 95% CI: 0.5–0.7, and OR: 0.8; 95% CI: 0.6–0.9 for those aged 12–14 and 15–17 years old, respectively). Teens with one risk behavior were more likely to be injured than those with no risk behavior (OR: 1.6; 95% CI: 1.3–2.1). Those with two or more risk behaviors were more likely to be injured (OR: 1.3; 95% CI: 1.1–1.6). Similarly, risk factors related to numbers of injured visits to outpatient services, were living in mountainous areas (OR: 1.4; 95% CI: 1.1–1.8), males (OR: 1.6; 95% CI: 1.4–1.8), oldest age group, and those who were with risk behaviors (OR: 1.3; 95% CI 1.1–1.6 for those with one risk behavior; OR: 1.4; 95% CI 1.2–1.8 for those with more than two risk behaviors).

In addition to factors in logistic model and proportional odds model, the injury type was included to control for the severity of injury while comparing the medical cost in outpatient services. The results showed the average injury cost per year per person was NTD851.4 (32NTD~1USD) (Table 3). Teenagers who lived in offshore areas, aged 12–14 years, and suffered from other injuries were associated with lower injury cost. In contrast, those with open wounds, bone fractures, and males spent more money in outpatient care. The household income and protective devices using habits were not related to medical cost. Number of risk behaviors was not associated with the cost of injury after controlling injury types.

## Discussion

This was the first study that used national representative data to look at factors associated with teenagers' injury, and injury related outpatient visits and costs, by regions of Taiwan. In general population, the nonfatal injury in teenagers was around 30%. The prevalence was 10% higher in mountains areas. The scope of the problem is not trivial. In Taiwan, the extensive motorcycle use can be a problem among teenagers. Compared with the other six major Asian countries, Taiwan had the highest rate in numbers of motorcycle per 1,000 persons (532 motorcycles/1000 persons) and per kilometer of road (327 motorcycles/kilometer of road) [17]. An observational study in Taiwan found that males had significantly higher risk of traffic injuries in all age by gender groups [18]. Motorcycle safety should be our first focus to reduce teenagers' injury.

Geographic locations of Taiwanese teenagers were significantly associated with injury and the use of injury-related outpatient services. In addition, males, older teens, and those with risk behaviors were significantly associated with injury. The results pointed to the universal problem, that was people with scarce resources and hazardous living environment were subject to injury, especially those with risk propensity.

Males were 1.3 times more likely to be injured. Once they were injured, they spent significantly more in outpatient services (Table 2). It is well known that males tend to engage in more dangerous activities and lack of self-protection skills. Previous studies have reported that males were more subject to injury than females [1,15,18]. An observational study found that males had significant higher risk of head injuries among those who were younger than 18 years old in Taiwan [19]. Age has been reported as an important factor for unintentional injuries in teenagers [20]. A cross-sectional study by Peter et al., who sampled more than 17,000 children and adolescents, reported that the annual injury rate for children 0–17 years old increased with age. Also, boys experienced significantly higher injury rates than girls, and adolescents aged 14–17 years experienced the highest overall rate and proportion of serious injuries [21]. Older male teens were also more likely to take greater risks [22]. The saying that adolescence is a time of learning and testing has been proved again in this study.

Socioeconomic factors such as education levels and household income were related to injury worldwide [5,23]. A study reported an evident relation between poverty and injury, because children with lower socioeconomic status generally live in un-safe environments, and face more danger, which increases the probability to injury [24]. A population-based study in Canada, which focused on about 96,000 children aged 0–10 years, also reported that children who had received social welfare at some time were at greatest risk [25]. The household income in our study was less important than in other countries. The respondents might have reported false incomes, or perhaps the variation in income level was too slight to show impact. In any event, teenagers were old enough to take risks regardless of family income. On the other hand, the living area of this study could be a proxy for socioeconomic status, since people lived in remote areas had access to little resources in terms of education or health care services.

We found a dose response relationship in number of risk behaviors and chance to be injured. For the injured, higher number of risk behaviors also related to higher number of outpatient visits. The pattern was consistent with other studies [26]. Literature showed that smokers appeared 1.5 times more likely than non-smokers to have motor vehicle crashes, 1.4–2.5 times more likely to be injured at work, and 2.0 times more likely to suffer other unintentional injuries [27]. Except for smoke, a strong relationships between drinking and traffic crashes have also been reported [28,29]. Drunk driving (or riding motorcycle) was the first leading cause of death in traffic crashes of Taiwan, and most of the riders aged between 18 and 24 [30].

This study had several limitations. First, the NHI medical claim data did not identify the causes and severity of injuries, the time the injury occurred, or weather factors. These factors would be useful for designing a prevention program. Stratifying the remote samples to more levels for analysis was difficult due to the small sample sizes. Finally, many conditions in remote areas were different from the general area of Taiwan. For example, police manpower in remote areas was lower than in the general area of Taiwan. If most injuries resulted from traffic accidents, the difficulty of enforcing laws must be taken into account [31].

Differences in medical choices also need to be considered. Some mountain people would rather choose alternative medicine than see a doctor. This could cause the underestimation of injury in remote areas.

## Conclusion

This study found that around 30% of the teenagers were injured in a year, not including the dead. Each of the injured spent at least 851.4NTD (~27USD) for outpatient visits in a year. The scope of the problem was not trivial. Teenagers in mountainous areas had the highest injury prevalence. The major risk factors were gender, age, and risk behaviors. Limited medical resources, risky behaviors, and hazardous environments were the reasons for high prevalence but low medical cost in remote areas. Putting information on mortality, prevalence, and medical expenditure together, we speculate that mountainous areas' teens are more subject to fatal or severe injury than minor injuries. The outpatient cost of injury only reflected the conditions of teenagers' minor injury. The severe injuries need more hospitalization data to analyze. Increasing the medical facilities and personnel in mountainous areas would be impractical, even though medical resources are very often limited [32].

One way to decrease the injury rates in those areas is to promote safety and behavioral changes through targeted education or community-based programs. Another is to improve transportation of patients from remote areas for medical treatments, by such means as helicopters. Legislation is the most effective way to reduce accidents. For example, enforcing alcohol consumption below certain levels for driving has reduced fatal accidents by 10% to 30% in teenagers [33-35]. The Taiwanese government has enforced the laws for wearing helmets for riding motorcycles and seatbelts for driving automobiles. However, people in remote areas were at the lowest rate of observing those laws [36]. Empower the local people to design education and prevention programs according to their needs and culture is recommended.

## Competing interests

The author(s) declare that they have no competing interests.

## Authors' contributions

HY Chen, main research assistant of the study. He managed and cleaned the data set, analyzed the data for this study, and drafted the manuscript.

HY Chang, principle investigator of the study, involved in all stages of the project and revised the whole manuscript few times. She also coordinated the National Health Interview Survey.

SF Shih and CC Hsu both contributed in the intellectual discussion of the concept of the article and the idea of data analysis.

YH Lin, conducted the fieldwork of the National Health Interview Survey and contributed to the intellectual discussion of the concept of the article.

YT Shih, lead investigator, involved in coordinating the 2001 National Health Interview Survey and contributed to the intellectual discussion of the concept of the article.

## Pre-publication history

The pre-publication history for this paper can be accessed here:



## Figures and Tables

**Table 1:** The injury prevalence of study sample by characteristics from log-linear model

		Injury					
Variables	Categories	N	Yes	%	Adjusted residual	Significance	95%CI

Main variable							
Area	Offshore	177	61	34.5	1.1	28.18	40.82
	Mountain	220	90	40.9	3.4 *	33.61	48.19
	Taiwan	2752	819	29.8	-3.3*	5.81	53.79
Control Variables							
Age	12~14	637	144	22.6	-5.0*	12.04	33.16
	15~17	882	246	27.9	-2.2*	14.58	41.22
	18~21	1630	580	35.6	6.0*	16.27	54.93
Gender	male	1606	558	34.7	-4.9*	15.62	53.78
	female	1543	412	26.7	4.9*	9.32	44.08
Education	junior	1075	277	25.8	-4.4*	11.45	40.15
	senior	1404	493	35.1	4.7*	17.22	52.98
	college	670	200	29.9	-0.6	18.05	41.75
Income (NTD)	<30,000	625	189	30.2	-0.4	18.72	41.68
	3~49,999	774	232	30.0	-0.6	17.25	42.75
	5~69,999	839	271	32.3	1.1	18.76	45.84
	7~99,999	480	155	32.3	0.8	22.05	42.55
	>100,000	429	123	28.7	-1.0	19.33	38.07
Risk behaviors	None	2494	684	27.4	-8.0*	5.13	49.67
	one	396	170	42.9	5.6*	33.05	52.75
	two or more	259	116	44.8	5.1*	36.78	52.79
Seatbelt	Yes	1780	575	32.3	2.1*	12.57	52.03
	No	1369	395	28.9	-2.1*	12.09	45.62
Helmet	Yes	2641	838	31.7	2.6*	7.81	55.65
	No	508	132	26.0	-2.6*	16.10	35.87

Rate based on NHI of OPD records; N: total sample in NHIS;

Income: household income in NTD (32NTD ≅ 1USD) *: adjusted residual >| 1.96|

**Table 2:** Risk factors associated with injury (logistic) and number of injury visits (proportional odds model)

Odds Ratio Estimation						
Model	Logistic			Proportional odds		

Effect	Odds	95% CI		Odds	95% CI	
Intercept 1	---			0.1	--	
Intercept 2	---			0.2	--	
Area (vs Taiwan)						
Offshore	1.4	0.98	1.89	1.0	0.79	1.30
Mountain	1.7*	1.27	2.30	1.4 *	1.14	1.76
Gender (vs female)						
Male	1.3*	1.12	1.55	1.6*	1.41	1.82
Age (vs 18–21)						
12–14	0.6*	0.46	0.70	0.7*	0.57	0.79
15–17	0.8*	0.64	0.94	0.9*	0.75	1.00
Risk behavior (vs None)						
One	1.6*	1.30	2.06	1.3*	1.13	1.58
Two or more	1.3*	1.12	1.55	1.6*	1.41	1.82
Household Income (vs <29,999)						
30,000~49,999	1.0	0.76	1.22	1.0	0.79	1.14
50,000~69,999	1.1	0.89	1.41	1.1	0.88	1.26
70,000~99,999	1.1	0.86	1.46	1.0	0.82	1.24
>100,000	0.9	0.68	1.19	1.0	0.79	1.21
Seatbelt (vs Yes)						
No	0.9	0.79	1.10	1.0	0.92	1.19
Helmet (vs Yes)						
No	0.9	0.73	1.16	0.9	0.74	1.07

*: p < 0.05

Intercept 1: the highest group versus middle or the lowest group for people who lived in Taiwan, female, 18–21, and non-smokers

Intercept 2: the highest or middle group versus the lowest group for people lived in Taiwan, female, 18–21, and non-smokers

**Table 3:** Factors associated with logarithm of injury cost by mixed model

	Estimate	Cost^1^	95% C I		p-value
Intercept	6.7	851.4	6.57	6.92	<.0001
Area (vs Taiwan)					
Offshore	- 0.4	545.8	-0.61	-0.28	<.0001
Mountain	0.0	825.3	-0.19	0.12	0.691
Gender (vs Female)					
Male	0.2	1012.5	0.07	0.27	0.001
Age (vs 18–21)					
12–14	- 0.2	721.5	-0.29	-0.04	0.012
15–17	- 0.1	776.0	-0.20	0.02	0.098
Risk behavior (vs None)					
One	- 0.1	767.9	-0.25	0.04	0.165
Two or more	0.0	821.4	-0.17	0.09	0.588
Household Income (vs <29,999)					
30,000~49,999	- 0.1	750.5	-0.27	0.02	0.090
50,000~69,999	- 0.1	797.2	-0.21	0.08	0.377
70,000~99,999	0.1	913.9	-0.10	0.24	0.407
>100,000	- 0.1	807.6	-0.22	0.11	0.534
Seatbelt (vs Yes)					
No	- 0.1	777.5	-0.22	0.04	0.172
Helmet (vs Yes)					
No	0.0	829.6	-0.12	0.07	0.596
Injury types (vs Sprains/Strains)					
Open Wounds	0.6	1579.1	0.50	0.73	<.0001
Fractures	0.5	1382.2	0.27	0.70	<.0001
Contusions	- 0.1	788.9	-0.18	0.03	0.153
Other Injuries	- 0.4	577.7	-0.52	-0.26	<.0001

Cost^1^: cost was calculated as exp (intercept + coefficient)
